# Neurological outcomes in Chiari type II malformations and their correlation to morphological findings and fetal heart rate patterns: a retrospective study

**DOI:** 10.1186/s13104-015-1014-2

**Published:** 2015-02-27

**Authors:** Yuka Otera, Seiichi Morokuma, Kotaro Fukushima, Ai Anami, Yasuo Yumoto, Yushi Ito, Masayuki Ochiai, Kimiaki Hashiguchi, Norio Wake, Haruhiko Sago, Kiyoko Kato

**Affiliations:** Center for Maternal-Fetal and Neonatal Medicine, National Center for Child Health and Development, Tokyo, Japan; Department of Obstetrics and Gynecology, Kyushu University Hospital, Kyushu University, 3-1-1 Maidashi, Higashi-ku, Fukuoka, 812-8582 Japan; Comprehensive Maternity and Perinatal Care Center, Kyushu University Hospital, Fukuoka, Japan; Department of Neurosurgery, Graduate School of Medical Sciences, Kyushu University, Fukuoka, Japan; Research Center for Environmental and Developmental Medical Sciences, Kyushu University, Fukuoka, Japan; Department of Obstetrics and Gynecology, Graduate School of Medical Sciences, Kyushu University, Fukuoka, Japan

**Keywords:** Chiari type II malformations, Fetal heart rate pattern, Quiet phase, Neurological outcomes

## Abstract

**Background:**

Correlations among Chiari type II malformation (CMII) morphological findings, the proportion of fetal heart rate patterns corresponding to the quiet phase (QP), and neurological outcomes have yet to be investigated.

**Findings:**

The correlations among the morphological findings (i.e., the degree of ventriculomegaly, myelomeningocele levels, and degree of cerebellar herniation), proportion of time spent in QP, and developmental quotients (DQs) were analyzed in 22 children. The proportion of time spent in QP was compared between children with poor neurological outcomes (n = 9) and those with good outcomes (n = 13). Pearson’s correlations and the Mann–Whitney *U*-test were used to assess for statistical significance; *P* < 0.05 was considered statistically significant. No significant differences were observed between the DQs and morphological findings, but the DQs and the proportion of time spent in QP were significantly correlated (r = 0.287, *P* = 0.01). The proportion of time spent in QP was significantly different between children with poor outcomes and those with good outcomes (median, 11% [range, 0–32%] vs. 28% [range, 2–55%]; *P* = 0.006).

**Conclusions:**

The proportion of fetal heart rate patterns corresponding to the QP might be a useful predictor of neurological outcomes in 2-year-old children with CMII.

## Findings

### Background

Chiari type II malformations (CMIIs), present as the descent of both cerebellar tonsils and vermis below the foramen magnum, are associated with myelomeningocele [1]. Morphological findings such as the level of the myelomeningocele and the degree of hydrocephalus have been reported to correlate with neurodevelopmental outcomes [2,3]. However, Vossen et al. reported that the morphological findings are not predictive of long-term neurodevelopmental outcomes [4]. The lack of a clear correlation between the morphology and long-term outcomes has resulted in ongoing controversy.

Mirmiran et al. reported that a significant relationship exists between abnormal cerebral ultrasonography findings and behavioral development in infants [5]. Previous studies on fetal behavioral states have found that each state is associated with a typical fetal heart rate pattern and that the linkage between these variables is so strong that the different states can be recognized solely by monitoring the heart rate [6]. Abnormalities in fetal behavioral development, recognized by different heart rate patterns, may also be related to abnormalities in brain development. However, such examinations have not been previously conducted.

In this study, we determined whether the heart rate patterns and/or morphological findings are related to long-term neurodevelopmental outcomes by investigating the correlations among the morphological findings, proportion of each fetal heart rate pattern, and neurological developmental outcomes in CMII.

### Methods

This retrospective study reviewed CMII-complicated, singleton pregnancies with normal karyotypes between January 1998 and October 2012 at the Kyushu University Hospital (Fukuoka, Japan) and between January 2002 and December 2012 at the National Center for Child Health and Development (Tokyo, Japan). All newborns included in the study had been diagnosed with CMII using postnatal magnetic resonance imaging (MRI), and they were treated in the pediatric divisions of these two hospitals. Fifty-three newborns were enrolled, and 31 newborns were excluded because of a condition unrelated to CMII (2 newborns), preterm deliveries before 35 weeks of gestation (5 newborns), cardiotocography (CTG) recordings were <50 min (13 newborns), the absence of CTG recordings between 35–37 weeks of gestation (1 newborn) [7], or the absence of developmental outcome assessments between 18–24 months of age at the time of the enrollment (13 newborns). Three of 13 newborns with CTG recordings <50 min were also absent from the developmental outcome assessments. Ultimately, 22 newborns met all the inclusion criteria and were included in the analysis. In addition, we analyzed 10 newborns without adequate CTG recordings in order to determine whether there were any correlations between the morphological findings and DQs.

Three representative morphological findings (i.e., the degree of ventriculomegaly, level of the myelomeningocele, and degree of cerebellum herniation) were used as indicators for evaluating central nervous system malformation [2,3]. The degree of ventriculomegaly was evaluated by determining the atrial width of the lateral ventricle, measured in the axial plane, within 7 days of delivery. The myelomeningocele levels were ascertained using MRI scans taken within 2 days after birth and before surgical repair of the myelomeningocele [8]. The myelomeningoceles were defined according to the lowest intact vertebral segment and were characterized as thoracic, high lumbar (L1–2), mid-low lumbar (L3–4), low lumbar-high sacral (L–5 to S–l), or sacral [2]. The degree of cerebellum herniation was classified into three groups as follows: C2, below the foramen magnum to the inferior margin of C2; C3, below C2 to the inferior margin of C3; and ≥ C4, below C3 to the inferior margin of ≥ C4 in the sagittal plane of the MRI scan [8].

The fetal heart rate pattern was classified as corresponding to either the quiet phase (QP) or other. QP was characterized by a stable heart rate within an oscillation band of <10 beats/min [6,9]. All CTG recordings were obtained >35 weeks of gestation in 1 of 2 periods: 09:00–12:00 am or 02:00–04:00 pm, 2 h after the mother’s meal. Fetal tachycardia and decelerations were not related to non-reassuring fetal status in the CTG recordings for each fetus. Fetal heart rates were determined during each 1-min period by two examiners, and the periods with small oscillation bands were classified as QP only when the duration was longer than 3 min [9]. The QP proportion of the observation time (QP%) was used as the primary indicator in this study. In case of several CTG recordings after >35 weeks of gestation, the QP% was calculated as the proportion of the total QP time within the total observation time of the several CTG recordings.

Between 18–35 months of age, all the patients underwent developmental outcome assessments using the Enjoji Analytical Developmental Test [10]. This test has three major categories of DQ: motor, social, and speech. In the current study, only the social and speech DQs were used, as they represent mental development. The motor DQ was not used in order to avoid potential evaluation bias because of the inclusion of a motor DQ that reflects physical abilities, including skilled hand motor activities; CMII patients usually have motor dysfunction. Subjects with scores of ≥80 were considered to have normal mental development, and those with scores of <80 were considered to have abnormal mental development. Poor neurological outcomes were defined as DQs of <80 or a history of seizures [8,11]. Pearson’s correlations and the Mann–Whitney *U*-test were used to assess for statistical significance; *P* < 0.05 was considered statistically significant.

### Ethical considerations

We obtained approval from the Kyushu University Institutional Review Board for this observational study, and informed consent was obtained from the mothers of all the affected infants.

### Results

We compared the patients’ clinical characteristics, morphological findings, and neurological outcomes at approximately 2 years of age.

The gestational age at delivery ranged from 35 to 39 weeks, with a median age of 37 weeks. The median birth weight was 2,734 g (range, 1,910–3,320 g), and 68% of the patients were male newborns. One patient was born by transvaginal delivery, while the others were delivered by cesarean section. The median Apgar score was 8 at 1 min (range, 2–9 min) and was 9 at 5 min (range, 7–9 min). Eighteen patients had ventriculoperitoneal shunts, and 6 had shunt revisions. The median atrial width of the lateral ventricle was 22 mm (range, 10–37 mm). With regard to the myelomeningocele level, there were 5 thoracic, 1 high lumbar (L1–2), 4 mid-low lumbar (L3–4), 8 low lumbar-high sacral (L–5 to S–l), and 4 sacral cases. The patients were also classified based on their cerebellum herniation level, including 7 cases at C2, 4 at C3, and 11 at ≥ C4.

Seven patients had abnormal developmental quotients (DQs), and 3 experienced seizures, which were considered poor neurological outcomes [11]. Among these patients, 1 had both an abnormal DQ and experienced seizures. No significant differences in the patients’ clinical characteristics were noted between patients with poor neurological outcomes and those with good outcomes.

**Figure 1:**
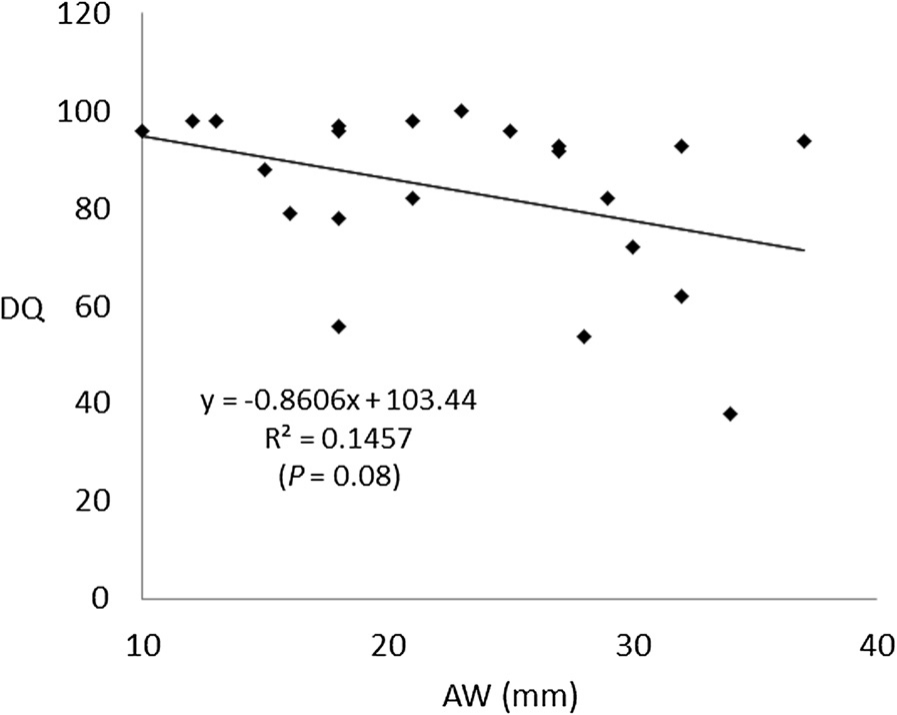
**Correlations between the developmental quotient (DQ) and atrial width (AW) of the lateral ventricle.**

**Figure 2:**
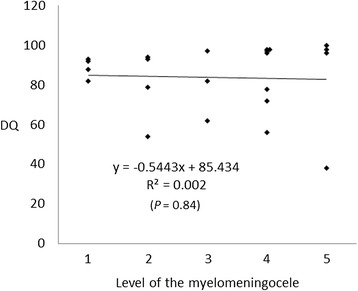
**Correlation between the developmental quotient and the level of the myelomeningocele.** 1, thoracic; 2, L1–2; 3, L3–4; 4, L5–S1; and 5, sacral.

**Figure 3:**
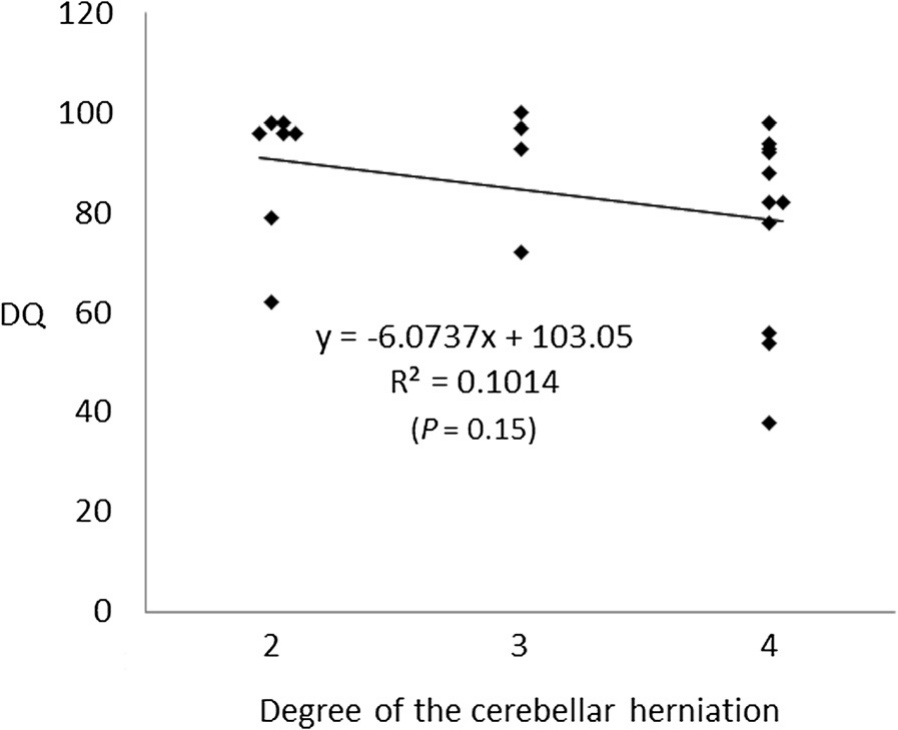
**Correlation between the developmental quotient (DQ) and the degree of the cerebellar herniation.** 2, below the foramen magnum to the inferior margin of C2; 3, below C2 to the inferior margin of C3; and 4, below C3 to the inferior margin of ≥ C4.

No significant correlations were also observed between the DQs and the degree of morphological findings (DQs vs. the degree of ventriculomegaly: r = 0.146, *P* = 0.08; DQs vs. the lesion level of the myelomeningocele: r = 0.002, *P* = 0.84; and DQs vs. the degree of cerebellar herniation: r = 0.101, *P* = 0.15) (Figures 1, 2 and 3). In addition, there were no significant correlations between the DQs and the degree of morphological findings among the 32 newborns, including the 10 excluded newborns with insufficient or no CTG recordings (DQs vs. the degree of ventriculomegaly: r = 0.070, *P* = 0.14; DQs vs. the lesion level of the myelomeningocele: r = 0.018, *P* = 0.46; and DQs vs. the degree of cerebellar herniation: r = 0.016, *P* = 0.49).

**Figure 4:**
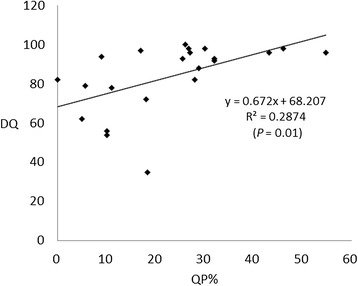
**Correlation between the developmental quotient (DQ) and the proportion of fetal heart rate patterns that correspond to the quiet phase (QP%).**

**Figure 5:**
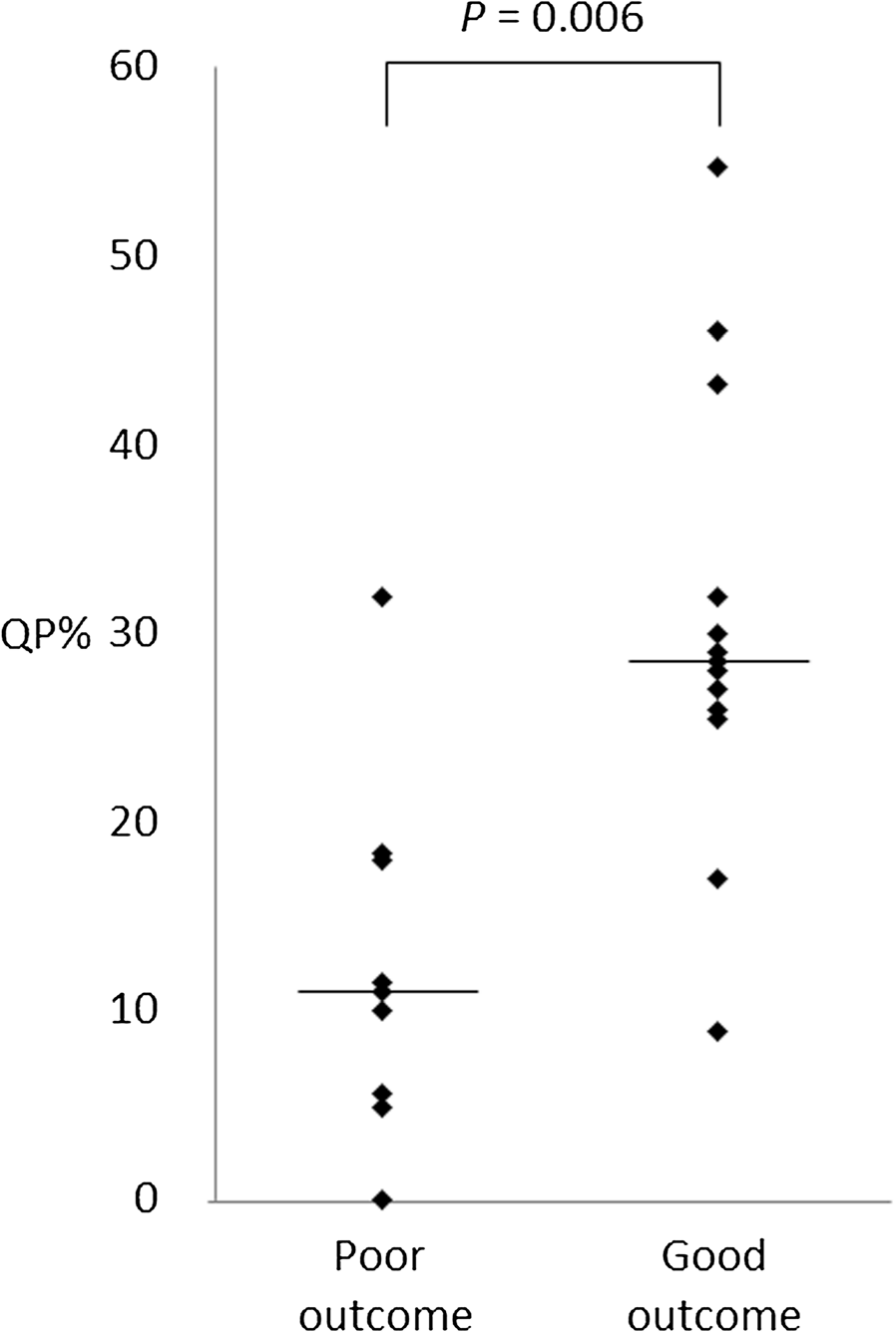
**Quiet phase proportions (QP%) between children with poor neurological outcomes and children with good outcomes.** The solid line indicates the median value for each group.

Figure 4 shows a significant correlation (r = 0.287, *P* = 0.01) between the DQs and the proportion of time spent in the QP among the newborns. Additionally, significant differences in the QP proportions were noted between patients with poor neurological outcomes (cases 1–9) and those with good outcomes (cases 10–22) (median, 11% [range, 0–32%] vs. median, 28% [range, 2–55%]; *P* = 0.006, Mann–Whitney *U*-test) (Figure 5).

No significant differences with regard to DQs were noted between patients with or without ventriculoperitoneal shunts or shunt revisions (*P* = 0.33 and *P* = 0.55, respectively).

### Discussion

In this study, the proportion of time in QP was significantly correlated with the DQ at 2 years of age; however, the morphological findings were not correlated with DQ. Coniglio et al. reported a negative correlation between the degree of prenatal ventriculomegaly and cognitive DQ, with an increasing severity of ventriculomegaly being associated with lower cognitive developmental outcomes. They also reported a positive correlation between the prenatal anatomic level of the myelomeningocele and the cognitive DQ; children with lower-level lesions demonstrated better cognitive outcomes [2]. Chao et al. reported that worsening cerebellar herniation on MRI was significantly associated with childhood seizure activity, a high risk of bladder dysfunction, and lack of independent ambulation [8]. However, Vossen et al. reported that none of the ultrasonography features, including the lesion level or ventriculomegaly, were predictors of motor or mental functioning at 5 years of age [3,4]. Similarly, in the current study, no significant correlations were noted between the degree of ventriculomegaly, myelomeningocele lesion level, or degree of cerebellar herniation and neurological outcomes. In a report by Vossen et al., although the lesion level was not a predictor of mental outcome, it was a predictor of survival. Morphological findings may not predict long-term neurological outcomes in extremely severe cases where survival is uncertain.

In the present study, the proportion of time that the heart rate was in QP was significantly correlated with the DQ at 2 years of age. Similarly, the proportion of time in QP was correlated with neurological prognoses, including clinical symptoms. The median proportion of time in QP among patients with good outcomes was 28%. This corresponds with the findings reported by Timor-Tritsch et al. (30.4%), suggesting that this proportion of time in QP is within the normal range [12].

Active sleep is considered a primitive sleep state that originates in the reticular activating system. However, quiet sleep requires more complex cortical integration and control [7,12]. Terao et al. reported that changes in fetal heart rates were correlated with anatomic defects in anencephalic infants. Anencephalic fetuses with a cortex showed a biphasic active-quiet pattern. Conversely, anencephalic fetuses with a midbrain but without the cortex did not show the biphasic pattern—only the active phase was exhibited [13]. Such observations imply that the cerebral cortex plays an important role in the genesis of quiet sleep. Among infants delivered at or >35–36 weeks of gestation, quiet sleep may be an indication of the functioning of the thalamocortical pathway [7]. The proportion of time in a particular sleep state is believed to predict normal development. In this study, neurological outcomes were examined only in children with CMII, because hydrocephalus has heterogeneous etiologies that make the combined examination of the various types of hydrocephalus difficult [14].

An indication of whether neurological impairment might develop would be significant in the management of neonates with this malformation. Although our study findings demonstrate that the morphological findings may not be useful for predicting long-term neurological outcomes in children with CMII, the proportion of time that a fetus spends in QP might be a useful indicator of future outcomes. However, these conclusions are limited by the small study size, and additional prospective studies are required to draw conclusions that are more concrete.

### Conclusions

Our findings showed that the proportion of heart rate patterns corresponding to the QP could be a useful predictor of neurological outcomes in 2-year-old children with CMII.

## Abbreviations

CMII: Chiari type II malformation
QP: Quiet phase
DQ: Developmental quotient
CTG: Cardiotocography
MRI: Magnetic resonance imaging
